# Supplementary material for: The OmpR-like Transcription Factor as a Negative Regulator of hrpR/S in Pseudomonas syringae pv. actinidiae
Source: Int J Mol Sci. 2022 Oct 14;23(20):12306. doi: 10.3390/ijms232012306 (PMC9602974; doi:10.3390/ijms232012306)
Supplement: Supplementary file 1 [file ijms-23-12306-s001.zip › R2--Supplementary Materials.pdf]

Supplementary Materials

Table S1. Primers used in this study

Primer pairs	Primer sequence (5'-3')	Product size (bp)
13375NO	GAGCTCGGTACCCGGGGATCCTGGCGATGGGCTTGCTG	910
13375NI	GCCAGGTTGACTGAGTTCGGTGACAATTTCCTT	
13375CO	GCCAAGCTTGCATGCCTGCAGTGACTGCTGCTGAATGCGATAA	870
13375CI	CCGAACTCAGTCAACCTGGCACCGCACAG	
13375F	GGAATTCCATATGACCCGAATTCTGGCAA	681
13375R	AAACTGCAGTTACAGATCCTCTTCAGAGATGAGTTTCTGCTCGACGGGTTCGGCAATGA	
13375-RT-F	GGCAGATAGTCATCGCCTCC	123
13375RT-R	CCTTACCATCGTGACCCACC	
SacB-F	GCAAACACTGGAACTGAAGATGG	478
SacB-R	TTCCTTTCGCTTGAGGTACAGC	
M13F	GTTTTCCCAGTCACGAC	1023
M13R	CAGGAAACAGCTATGAC	
Km12-F	TCACGAGCACTTCACCAACA	/
Km12-R	AATCTTATCTCATCCGCCAAAA	
Psa-F	CAGAGGCGCTAACGAGGAAA	311
Psa-R	CGAGCATACATCAACAGGTCA	
gyrA-RT-F	AACATTCCGCCGCATAACC	243
gyrA-RT-R	CTGACGACCGCCCACCTT	
gyrB-RT-F	ACCCGAACGAAGCCAAAGC	201
gyrB-RT-R	ATCCGCCAGCAGAGTCCC	
HrpS-F	CGGCACGGGCAAAGACA	269
HrpS-R	GCGCTCGGGTTTCCAACA	
HrpL-F	TGCTCAGGGCGTTTATCCA	130
HrpL-R	AGCCAGGTCTGCGGTTTACT	

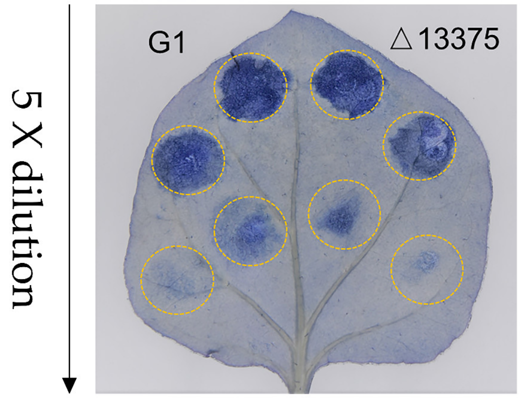

Figure S1. Deletion of *ompR-like* in *Pseudomonas syringae* pv. *actinidiae* (*Psa*) did not affect its ability to induce hypersensitive reaction (HR) on non-host *Nicotiana benthamiana* leaves. Bacterial suspensions of the wildtype *Psa* strain G1 and the *ompR-like* deletion mutant (Δ13375) were infiltrated into *N. benthamiana* leaves. The initial bacterial concentration was 5 × 10^7 CFU/mL. The leaves were stained with trypan-blue for HR observation after 24 h.

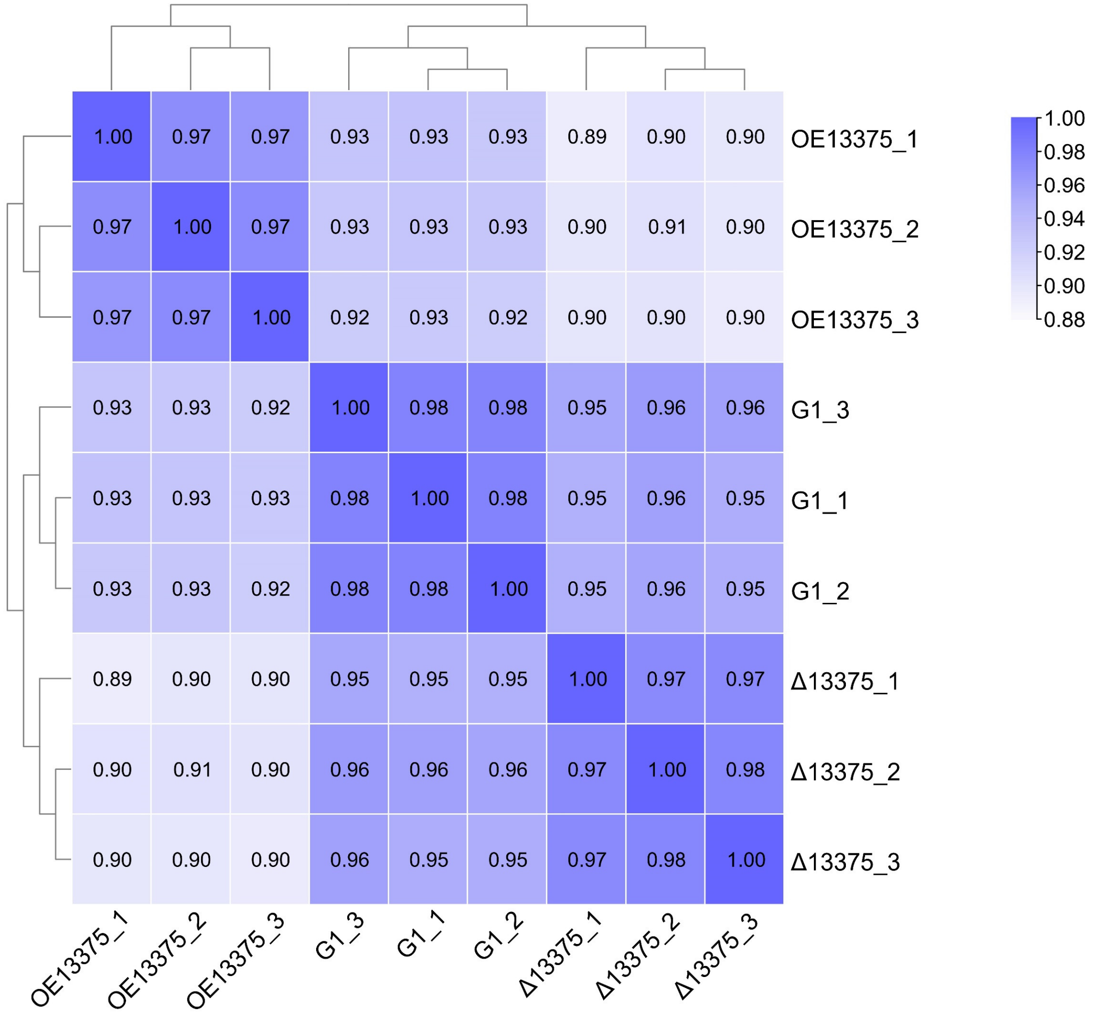

Figure S2. The correlations between RNA-seq samples. Three independent samples were sequenced for each strain. G1: wild-type strain; Δ13375: *OmpR-like* deletion mutant; OE13375: *OmpR-like* overexpression mutant.

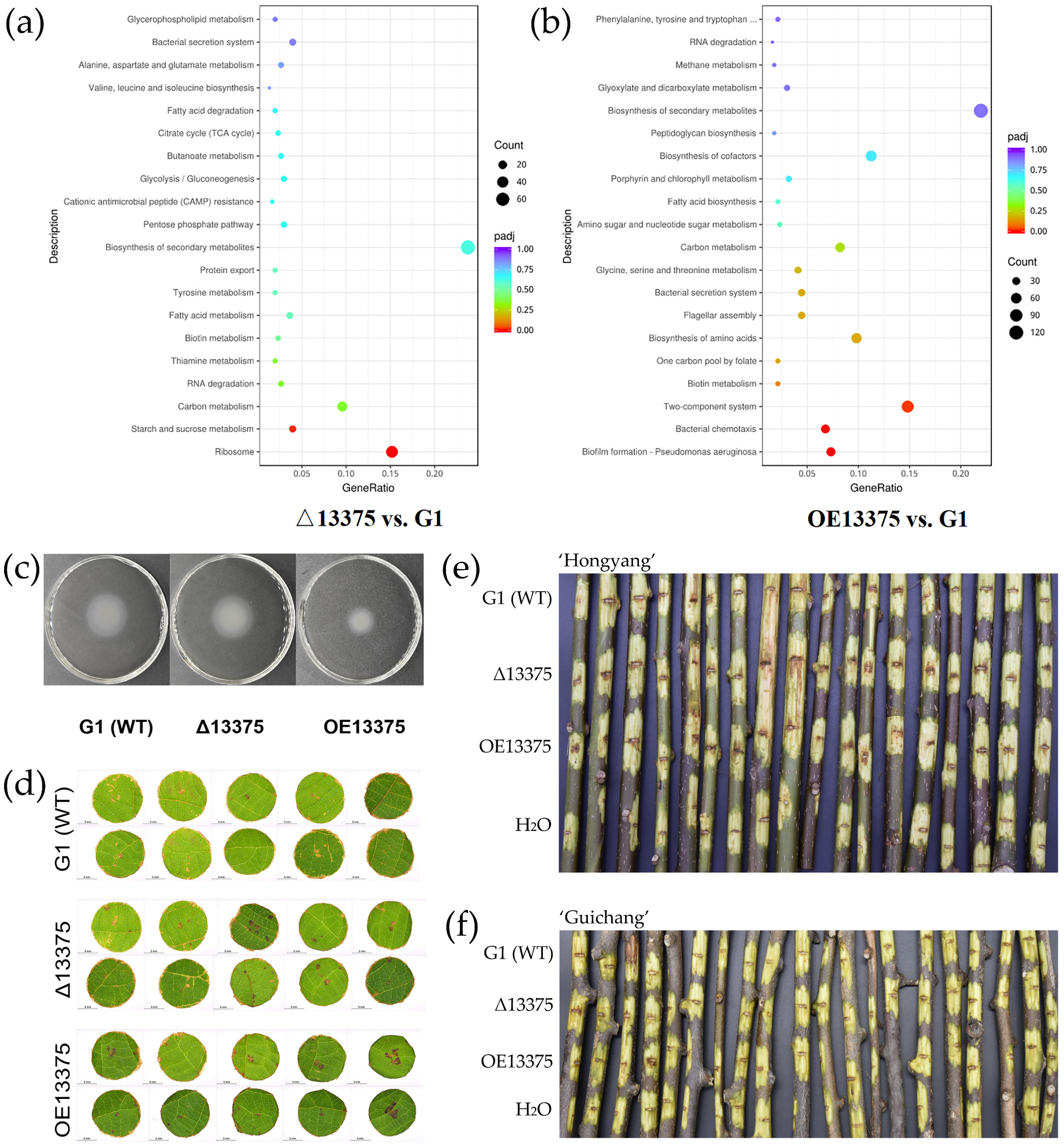

Figure S3. The OmpR-like transcription factor is also involved in other *Psa* biological pathways. (a) The enriched KEGG pathway of DEGs in Δ13375; (b) The enriched KEGG pathway of DEGs in OE13375; (c) The observation of swimming motility of G1, Δ13375 and OE13375 when strains with the same $OD_{600\ nm}$ values were titrated on KB plates containing 0.3% agar and incubated within 24 h at 25 °C; (d) The symptom for the inoculation assay on kiwifruit leaf discs as described in Fig. 4h. (e, f) The symptom for the inoculation assay on kiwifruit shoots as described in Fig. 4i. 'Hongyang', *Actinidia chinensis* var. *chinensis* cultivar 'Hongyang'; 'Guichang', *A. chinensis* var. *deliciosa* cultivar 'Guichang'. G1: wild-type strain of *Pseudomonas syringae* pv. *actinidiae*; Δ13375: *OmpR-like* deletion mutant; OE13375: *OmpR-like* overexpression mutant.

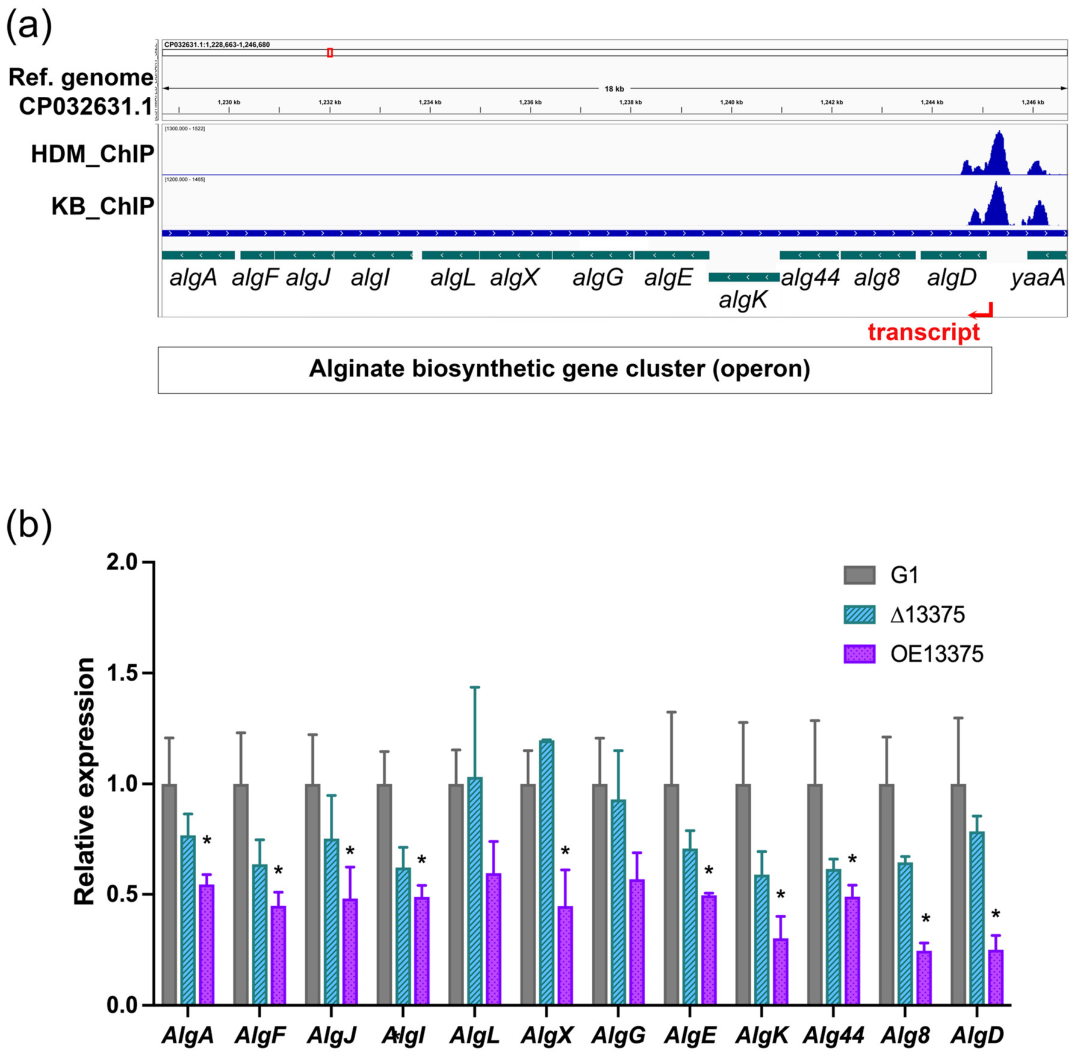

Figure S4. The alginate synthesis was regulated by OmpR-like. (a) The binding site of OmpR-like was detected upstream of *alg* operon. (b) All genes in the *alg* operon were downregulated in the OE13375 mutant, compared to the wildtype G1 strain. The difference was determined by the Student's *t* test (*, $P < 0.05$).

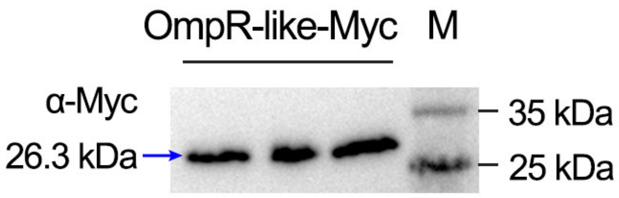

Figure S5. Western blotting detected the expression of OmpR-like-Myc (26.3 kDa) in OE13375 with the addition of 1 mM IPTG. Three samples (indicated in three lanes) were detected with α-Myc antibody. M, the molecular marker.
